# Supplementary material for: The Role of Visual Information Quantity in Fine Motor Performance
Source: J Funct Morphol Kinesiol. 2024 Dec 11;9(4):267. doi: 10.3390/jfmk9040267 (PMC11676225; doi:10.3390/jfmk9040267)
Supplement: Supplementary file 1 [file jfmk-09-00267-s001.zip › jfmk-3312449-supplementary.pdf]

Table S1. Quantity of visual information (points displayed).

	3 pts	6 pts	9 pts	12 pts	21 pts	30 pts	60 pts	120 pts	Full
**absolute error (mm)**	6.64 ± 1.78	5.46 ± 1.11	4.40 ± 1.26	3.77 ± 1.04	3.35 ± 1.12	3.29 ± 0.92	3.18 ± 0.99	2.86 ± 1.02	2.82 ± 0.92
**time of execution (s)**	12.68 ± 5.13	14.10 ± 5.35	14.47 ± 5.54	16.61 ± 5.63	17.52 ± 6.77	17.29 ± 7.44	17.32 ± 7.65	19.33 ± 8.91	20.85 ± 9.00
**speed (mm/s)**	99.28 ± 34.72	86.93 ± 29.83	82.22 ± 27.73	70.00 ± 22.37	71.91 ± 25.74	70.17 ± 24.92	66.27 ± 23.83	61.26 ± 25.15	57.19 ± 20.51
***smoothness (mm^2/s^6)**	4.17 ± 4.53	3.14 ± 4.46	2.47 ± 2.42	1.32 ± 6.45	1.53 ± 1.74	1.33 ± 1.06	1.25 ± 1.14	0.89 ± 0.83	0.80 ± 0.72
**pressure (a.u.)**	70.35 ± 13.97	71.26 ± 14.09	73.19 ± 14.93	69.39 ± 13.14	70.49 ± 13.95	67.58 ± 11.80	71.32 ± 14.37	72.78 ± 12.99	74.39 ± 11.72

* Smoothness value × 10^{-6}

[1] a.u. = arbitrary units
